# NPY and Gene Therapy for Epilepsy: How, When,... and Y

**DOI:** 10.3389/fnmol.2020.608001

**Published:** 2021-01-22

**Authors:** Stefano Cattaneo, Gianluca Verlengia, Pietro Marino, Michele Simonato, Barbara Bettegazzi

**Affiliations:** ^1^Vita-Salute San Raffaele University, Milan, Italy; ^2^San Raffaele Scientific Institute, Milan, Italy; ^3^Department of Neuroscience and Rehabilitation, Section of Pharmacology, University of Ferrara, Ferrara, Italy; ^4^Department of Medical Sciences, Section of Pediatrics, University of Ferrara, Ferrara, Italy

**Keywords:** viral vectors, epilepsy, gene therapy, Y2 receptor, NPY

## Abstract

Neuropeptide Y (NPY) is a neuropeptide abundantly expressed in the mammalian central and peripheral nervous system. NPY is a pleiotropic molecule, which influences cell proliferation, cardiovascular and metabolic function, pain and neuronal excitability. In the central nervous system, NPY acts as a neuromodulator, affecting pathways that range from cellular (excitability, neurogenesis) to circuit level (food intake, stress response, pain perception). NPY has a broad repertoire of receptor subtypes, each activating specific signaling pathways in different tissues and cellular sub-regions. In the context of epilepsy, NPY is thought to act as an endogenous anticonvulsant that performs its action through Y2 and Y5 receptors. In fact, its overexpression in the brain with the aid of viral vectors can suppress seizures in animal models of epilepsy. Therefore, NPY-based gene therapy may represent a novel approach for the treatment of epilepsy patients, particularly for pharmaco-resistant and genetic forms of the disease. Nonetheless, considering all the aforementioned aspects of NPY signaling, the study of possible NPY applications as a therapeutic molecule is not devoid of critical aspects. The present review will summarize data related to NPY biology, focusing on its anti-epileptic effects, with a critical appraisal of key elements that could be exploited to improve the already existing NPY-based gene therapy approaches for epilepsy.

## NPY Discovery, Evolution, and Function

Described in 1982, neuropeptide Y (NPY) is a 36-aminoacid peptide that shares high homology with its family members pancreatic peptide (PP) and peptide YY (PYY). The NPY ancestral gene appeared in vertebrates, evolving from an ortholog NPY-like system that regulates energy homeostasis in invertebrates acting on growth and reproduction (De Jong-Brink et al., 2001; Kooijman and Troost, 2007; Gershkovich et al., 2019). The family of Y peptides probably originated through a chromosome quadruplication event that took place during jawed vertebrate emergence (Larhammar and Salaneck, 2004).

NPY has a widespread expression throughout the central (CNS) and peripheral nervous system (PNS) and it is typically co-released with other neurotransmitters. An unusually broad repertoire of receptor subtypes mediate its actions, each activating specific signaling pathways in different tissues and cellular sub-regions (Leblanc et al., 1987; Keast, 1991; Dumont et al., 1992; Elfvin et al., 1997; Cerdá-Reverter and Larhammar, 2000; Wai et al., 2004).

During evolution, the NPY-like system has increased the complexity of its actions, with effects that in humans range from cell proliferation to the control of energy metabolism, pain and neuronal activity (Kuo et al., 2007; Tilan and Kitlinska, 2016). NPY is involved in cardiovascular and metabolic diseases, as well as in respiratory and neurologic disorders (Pedrazzini et al., 2003; Vezzani and Sperk, 2004; Atanasova and Reznikov, 2018), acting as a paracrine hormone in the periphery and behaving like a neuromodulator in the CNS.

In the CNS, NPY exerts its modulatory action both at cellular (excitability, neurogenesis) and at circuit level (food intake, stress response, and pain perception). It is expressed in different areas of the brain, from the neocortex to the posterior root of spinal nerves, usually in GABAergic interneurons, but also in long projecting catecholaminergic neurons; e.g., in the brainstem and in certain hypothalamic nuclei (Chronwall et al., 1985; de Quidt and Emson, 1986; Silva et al., 2005a; Benarroch, 2009). In the mesial temporal lobe, NPY is widely expressed in different subnuclei of the amygdala, where it is thought to exert a potent anxiolytic effect (Tasan et al., 2010; Wood et al., 2016), and in the hippocampus, where it displays an inhibitory action on excitatory synaptic transmission, mostly by reducing glutamate release (Colmers et al., 1985; Klapstein and Colmers, 1992; Greber et al., 1994; Mcquiston and Colmers, 1996). It is worth noting that, coherently with its homeostatic role, NPY projecting neurons are also close to circumventricular organs and sensory/secretory blood-brain interfaces (Wagner et al., 2015).

## Gene Structure

The human NPY gene (~8 kb) is located on chromosome 7p15 (genomic coordinates (GRCh38): 7:24,284,189-24,291,861). Regulatory elements have been found within 530 bases from the transcription start site and further regulatory sequences enhancing transcription and mRNA stability may be present up/downstream that region or even inside introns (Waldbieser et al., 1992; Waschek, 1995; Zhou et al., 2008). Single nucleotide polymorphisms (SNPs) in the coding region may increase NPY synthesis (Mitchell et al., 2008). The full length mRNA is 551 bp long (Minth et al., 1984). After translation in the endoplasmic reticulum, upon signal peptide truncation, NPY is directed to the secretory pathway.

## Peptide Trafficking, Processing and Release

**Figure 1:**
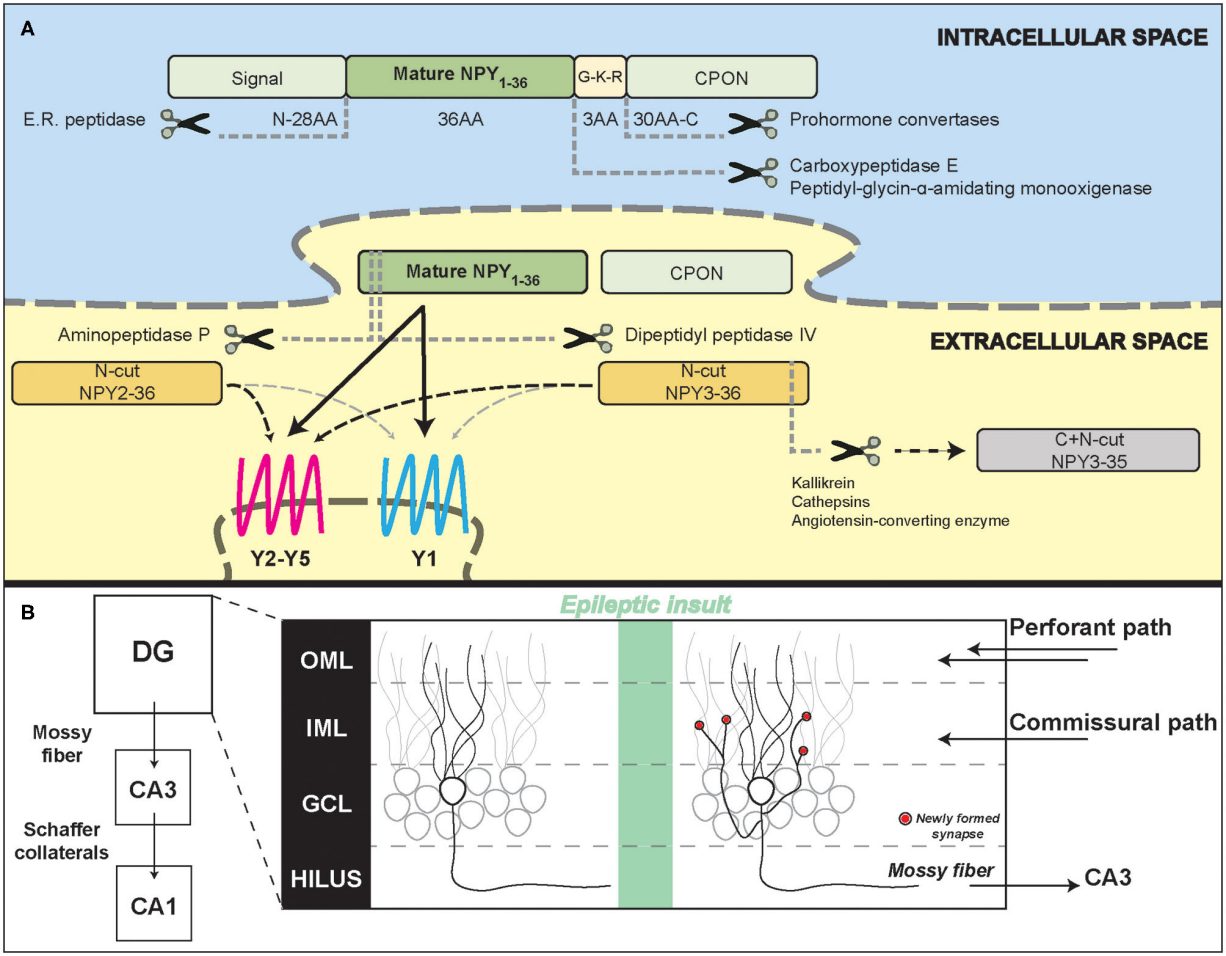
Neuropeptide Y processing and its potential role in the epileptic hippocampal network. **(A)** Schematic representation of NPY intracellular processing and extracellular metabolism. **(B)** Illustration of hippocampal formation rearrangements after an epileptic insult. Red dots represent synapses newly formed by the mossy fiber sprouting in the inner molecular layer that contain NPY and pre-synaptic Y2 receptors. DG, dentate gyrus; CA3/CA1, Cornu Ammonis; OML, outer molecular layer; IML, inner molecular layer; GCL, granule cell layer.

While trafficking inside dense core vesicles (DCVs), the full coding sequence of NPY, prepro-NPY, is sequentially split into three fragments (Figure 1A): (1) an N-terminus 28-amino acid (aa) signaling peptide, (2) the mature 36 aa, 4.2 kDa, peptide (NPY_1−36_), and (3) a 30-aa C-terminal flanking peptide of neuropeptide-Y (CPON). A glycine-lysine-arginine (G-K-R) site in proximity of the C-terminus of the mature 36 aa peptide is crucial for CPON cleavage by pro-hormone convertases and for the amidation of the mature NPY, performed by carboxypeptidase E and peptidyl-glycin-α-amidating monooxygenase. The CPON structure is highly conserved during evolution (Cerdá-Reverter and Larhammar, 2000). It has been suggested that it may play a role in epilepsy control, but current data do not confirm this hypothesis (Soud et al., 2019).

NPY and CPON containing DCVs are released upon calcium influx. The need of a long, high frequency firing rate for NPY release (Lundberg et al., 1986; van den Pol, 2012) has been questioned by evidence that NPY is released by hippocampal neurons even during physiological synaptic activity (Li et al., 2017).

## Metabolism

Once released in the extracellular space, mature NPY can bind to its receptors and activate signal transduction (Walther et al., 2011) or be metabolized, either close or far away from its release site, in the cerebrospinal fluid or in the blood. Proteolytic processing can alter the NPY signaling at either the N-terminal or C-terminal portion of the peptide and usually results in a modification of receptor binding affinity or inactivation followed by complete degradation, depending on a number of peptidases with compartment-dependent concentration and activity (Allen et al., 1987; Wagner et al., 2015).

The most common pathway of NPY metabolism is N-terminal cleavage by dipeptidyl peptidase IV (DP4) which is responsible for the formation of NPY_3−36_, followed by C-terminal processing by enzymes like kallikrein, cathepsins or angiotensin-converting enzyme (ACE) that in turn yield inactive NPY fragments. Aminopeptidase (AmP) instead produces NPY_2−36_, catalyzing a less efficient cleavage within the N-terminal region compared to DP4, which results in a lower relative concentration of this metabolite (Abid et al., 2009). Both NPY_3−36_ and NPY_2−36_ display a decreased affinity for Y1 receptors, therefore preferentially binding to other (Y2 and Y5) receptor subtypes (Grandt et al., 1996; Hubers et al., 2018; Yang et al., 2018).

After inactivation, other plasmatic peptidases catalyze the metabolism of smaller fragments, with the kidney playing a major role in residual NPY metabolism (Satoh et al., 1999). The estimated plasma half-life in human and animal studies is between 5 and 20 min (Pernow et al., 1986; Potter, 1987).

## NPY Receptors

The NPY system is not only multi-ligand, as described above, but also multi-receptor, and this makes it a complex target for therapeutic applications.

In fact, five different NPY receptors are expressed in mammals: Y1, Y2, Y4, Y5, and y6. While Y1, Y2, Y4, and Y5 are functional in all mammals, y6 is a pseudogene in humans and other primates and is missing also in the rat genome (Larhammar and Salaneck, 2004). NPY displays an especially high affinity for the Y1, Y2, and Y5 receptor subtypes: even if structurally different, these three receptors can respond to the same ligands. Y1 and Y4 form a receptor superfamily, while Y2 and Y5 have distinct, individual features (Larhammar and Salaneck, 2004). NPY receptors (YRs) have different affinities for the Y family hormone ligands, with Y4Rs binding preferably PP and Y2Rs binding NPY and N-terminally truncated peptides with similar affinity (Lindner et al., 2008). The genes encoding for NPY receptors are located on human chromosome 4 and probably arose by a duplication event from an ancestral NPY/PYY-binding receptor. All NPY receptors are widely expressed in the mammal brain, Y2 being the most abundant (Dumont et al., 1998). High levels of NPY binding can be revealed in the cortex, hippocampus, amygdala, striatum and cerebellum (Dumont et al., 1993).

Specific binding to Y1 receptors can be visualized in different layers of the cortex, in the CA1 and CA3 stratum radiatum, oriens, in the dentate gyrus of the hippocampus in the amygdala, striatum, cerebellum and, at lower levels, in some thalamic, hypothalamic and brainstem nuclei (Dumont et al., 1990, 1993; Aicher et al., 1991; Cabrele and Beck-Sickinger, 2000; Kopp et al., 2002). Outside the CNS, Y1Rs are also found in the adipose tissue and in vascular smooth muscle cells (Castan et al., 1993; Lindner et al., 2008). Y1Rs are mainly localized post-synaptically in neurons of the hippocampus (especially in CA3, CA1 and dentate gyrus), striatum and cortex (Wahlestedt et al., 1986; Caberlotto, 1997; Kopp et al., 2002), with a prominent somatic and dendritic localization (Kopp et al., 2002). However, some studies also suggest a pre-synaptic localization (Colmers et al., 1987, 1988; Flood and Morley, 1989; Pickel et al., 1998; Brumovsky et al., 2002; Glass et al., 2002; Kopp et al., 2002; Stanić et al., 2006; Li et al., 2017). Albeit NPY and Y1R scarcely co-localize (Stanić et al., 2011), the presence of Y1R on the cell soma of NPY-containing hilar interneurons and cultured hippocampal neurons is suggestive of a possible role of these receptors in an autoinhibitory feedback (St-Pierre et al., 2000; Paredes et al., 2003).

Together with Y5Rs, Y1Rs play an important role in regulating feeding behavior and energy homeostasis (Baldock et al., 2007; Nguyen et al., 2012). Y1R-mediated antidepressant and anxiolytic effects have been described in rodents (Wahlestedt et al., 1993; Verma et al., 2012), while the role in epilepsy remains controversial (see below). The anxiolytic effect of NPY in the basolateral amygdala has been attributed to the activation of Y1Rs (Sajdyk et al., 2004; Giesbrecht et al., 2010).

Y2Rs are expressed in many brain regions, including the hippocampus, thalamus, hypothalamus and cortex; in the peripheral nervous system, Y2Rs are found in parasympathetic, sympathetic and sensory neurons; finally, they are also present in the intestine and in certain blood vessels (Wahlestedt et al., 1986; Stjernquist and Owman, 1990; Gehlert et al., 1992; Dumont et al., 1993; Rettenbacher and Reubi, 2001). In the hippocampus, Y2 receptors are particularly enriched in the CA1 and CA3 areas, respectively in the stratum radiatum and in the pyramidal cell layer (Colmers et al., 1987, 1988, 1991; Monnet et al., 1992). Expression of Y1 and Y2 receptors is often complementary. For example, high levels of Y2Rs are detectable in the stratum oriens and radiatum of CA1-CA3, where Y1 receptor levels are relatively low, while the opposite is true in the dentate gyrus molecular layer (Stanić et al., 2011). Y2Rs are highly expressed in the terminal regions of mossy fibers and Schaffer collaterals (Jacques et al., 1997), where they act pre-synaptically by inhibiting calcium-mediated neurotransmitter release (Klapstein and Colmers, 1993). While NPY and a Y2R selective agonist inhibit evoked EPSPs on CA1 pyramidal cells, a Y2R selective antagonist is able to block the inhibitory action of NPY on glutamate release (El Bahh et al., 2002).

Y2Rs are expressed by both GABAergic and glutamatergic terminals (Stanić et al., 2006, 2011) and may therefore inhibit the release of both neurotransmitters, in particular under chronic epileptic conditions (Martire et al., 1993; Greber et al., 1994; Klapstein and Colmers, 1997; Vezzani and Sperk, 2004; Silva et al., 2005b). This makes Y2Rs an interesting target in epilepsy (Vezzani and Sperk, 2004). Y2Rs can also be localized along the course of axons in fiber tracts (in Schaffer collaterals, the fimbria and the stria terminalis (Dum et al., 2017)). These receptors are functionally coupled with G-protein signaling and show high affinity for their ligand (Dum et al., 2017), leaving open the possibility of a modulation through NPY volume transmission.

Y5Rs are mainly found in the hypothalamus and in the hippocampus (in the pyramidal cell layer of the CA2 region, with lower concentrations in the hilar region of the dentate gyrus and in the CA3 subregion), where they participate in the modulation of hippocampal excitability (Gerald et al., 1996; Dumont et al., 1998; Guo et al., 2002). Together with Y1Rs, Y5Rs contribute to the regulation of food intake and energy homeostasis, but they also display anticonvulsant effects (Woldbye et al., 1997; Criscione et al., 1998; Nanobashvili et al., 2004). Y5R KO mice display a reduced NPY-mediated inhibition of glutamatergic synaptic transmission and are therefore more susceptible to kainate-induced seizure mortality (Marsh et al., 1999; Baraban, 2004).

NPY receptors are G protein-coupled receptors (GPCRs) with seven transmembrane domains, acting preferentially *via* hetero-trimeric Gi/o proteins (Michel et al., 1998). They can trigger a variety of intracellular responses, including inhibition of adenylyl cyclase, regulation of potassium and calcium channels and activation of the mitogen-activated protein kinase (MAPK) cascade in some cell types (Howell et al., 2005; Lu et al., 2010; Thiriet et al., 2011; Shimada et al., 2012). Binding of the ligand to the receptor stabilizes an active receptor conformation, essential for inducing intracellular signal transduction. NPY binding modes vary with individual receptors, with different amino acids impacting anchoring, affinity and binding (Beck-Sickinger et al., 1994; Merten et al., 2007; Walther et al., 2012; Pedragosa-Badia et al., 2013; Kaiser et al., 2015; Yang et al., 2018). NPY peptides reach the receptors by lateral diffusion, after being pre-associated with the membrane through their C-terminal domain (Lerch et al., 2004; Thomas et al., 2005) that is also essential for the binding of NPY to specific receptors, in particular Y2 (Beck-Sickinger et al., 1994).

NPY receptors are predominantly expressed at the cell surface and sequence motifs essential for endoplasmic reticulum export and delivery to the membrane have been identified, particularly in the C-terminal portion of the protein (Walther et al., 2011, 2012). Y2Rs display desensitization (Ziffert et al., 2020a) but can undergo arrestin beta3-dependent and independent internalization only when exposed to high concentrations of agonist (Lundell et al., 2011; Walther et al., 2011). The low rate of Y2R internalization may depend on the presence of a N-terminal extracellular domain rich in acidic/anionic residues (Parker et al., 2001; Gicquiaux et al., 2002).

## NPY and Epilepsy

A consistent amount of data demonstrates the functional involvement of the NPY system in epilepsy. This statement is supported by two lines of evidence: (1) the epileptogenic process and epilepsy itself modify the expression pattern of the genes encoding NPY and its receptors; (2) acting as neuromodulators, NPY peptides control network excitability and homeostasis.

NPY expression is increased both in rodent and human hippocampal sections from temporal lobe epilepsy (TLE) surgical samples (Sperk et al., 1992; Furtinger et al., 2001), despite the strong loss of hilar GABAergic interneurons that physiologically express NPY. This is because the excitatory granule cells, which in epilepsy give rise to mossy fiber sprouting (MFS), have been demonstrated to ectopically produce and release NPY (Mathern et al., 1995; McCarthy et al., 1998). MFS, the aberrant sprouting of granular axons that recurrently innervate granule cell dendrites in the molecular layer generating an auto-excitatory loop (Figure 1B), is a marker of TLE, even if its pathophysiological role is still controversial (Cavarsan et al., 2018).

In patients with hippocampal sclerosis, another common pathological trait of TLE, a shift toward higher Y2 receptor density is observed in the CA1, CA3, in the hilar region and in the inner molecular layer of the hippocampus (Furtinger et al., 2001). This receptor up-regulation may support a persistent Y2R signaling, because it has been recently shown that Y1, but not Y2, receptors are rapidly internalized and recycled after binding to their ligand (Ziffert et al., 2020a,b). As noted above, increased Y2Rs signaling may imply an anti-epileptic effect (El Bahh et al., 2005). In fact, Y2R knockout mice are totally insensitive to the anti-epileptic actions of NPY, both *in vitro* and *in vivo* (Woldbye et al., 2005).

As opposed to Y2 receptor up-regulation in the epileptic hippocampus, it has been shown that Y1 receptor mRNA and binding actually decrease in kindled rats (Gobbi et al., 1998) and in intra-hippocampal kainate-treated mice (O'Loughlin et al., 2014). A reduced density of Y1Rs has been also demonstrated in human patients with hippocampal sclerosis, indicating a reduced expression of the receptor or a loss of Y1R-expressing neurons (Kofler et al., 1997; Furtinger et al., 2001). In addition, as mentioned above, Y1Rs are rapidly internalized after binding to NPY (Ziffert et al., 2020a,b). Y1R may be responsible of unfavorable effects in epilepsy, because administration of Y1R antagonists produces antiepileptic effects in animal models (Gariboldi et al., 1998; Vezzani et al., 2000) and Y1 KO mice display reduced mortality rate upon NPY administration (Lin et al., 2006). Thus, their reduced density and signaling may be interpreted as an antiepileptic adaptive mechanism. It cannot be excluded, however, that this adaptive downregulation could be linked to epilepsy-induced depressive or anxious behavior, described in patients and in animal models (Yilmazer-Hanke et al., 2016; Vrinda et al., 2017; Zanirati et al., 2018).

Similarly, the decreased density of Y5R in epilepsy models (Bregola et al., 2000) may represent a maladaptive alteration because the pharmacological activation of Y5Rs has been reported to exert antiseizure effects (Woldbye et al., 1997).

Expression levels of NPY-related genes may strongly vary across species, with rats having higher expression of both NPY and Y2 compared to mice (Nadler et al., 2007; Károly et al., 2015). Discrepancy between rodents and humans have been also found at the electrophysiological level. In human slices, prepared from surgically resected hippocampi of drug-resistant patients, NPY application reduces both lateral perforant path-evoked excitatory response in granule cells (Patrylo et al., 1999) and currents evoked by medial perforant path stimulation (Ledri et al., 2015). Conversely, experiments on hippocampal slices from an animal model of epilepsy (pilocarpine-treated rats) show that NPY does not affect the response of granule cells to perforant path stimulation but reversibly inhibits recurrent synaptic transmission of mossy fibers on granule cells themselves (Tu et al., 2005).

Even if the precise mechanism of action of the NPY system on the epileptic network has not been completely clarified, a clear effect of the neuropeptide in inhibiting epileptiform activity on human hippocampal sections challenged with [0] Mg^2+^/4-amino-piridine has been demonstrated (Wickham et al., 2019), further corroborating the idea that the anti-epileptic effect is predominantly mediated by Y2. It has been shown indeed that the effect of NPY administration can be abolished by treatment with a specific Y2 receptor antagonist (Tu et al., 2005; Ledri et al., 2015; Wickham et al., 2019).

An epileptic insult in the brain can result in a synchronous activation of granule cells that fail to inhibit the propagation of excitation from the entorhinal cortex to the hippocampus. Subsequent compensation mechanisms might arise, and it is tempting to speculate that granule cells, with the death of their target inhibitory neurons, sprout their axons to the molecular layer, increasing excitability but, at the same time, producing synapses containing both NPY and Y2R at the presynaptic level. Within this view, NPY would act as a compensatory negative feedback, activated upon high frequency stimulation, where NPY is released from granular axons and reduce the overall hyperactivity of the local neuronal network. This hypothesis is also in line with the discrepancies that have been observed between mice and rats, with the latter showing higher recurrent mossy fiber sprouting and displaying higher levels of NPY and Y2 immunoreactivity coupled with a stronger inhibitory effect upon NPY application (Tu et al., 2005).

Taken together, these data suggest a significant involvement of NPY in the epileptogenic process, supporting the idea that both pharmacological and genetic approaches targeting the NPY system may represent effective strategies for the treatment of epilepsy. In the frame of this article, we will focus on the latter (gene therapies).

## Exploiting NPY in Gene Therapy

In the last two decades, a great effort has been devoted to the development of gene therapy products for life-changing treatments in epilepsy. In that context, one of the most prominent strategies has been the direct infusion in epileptogenic areas of recombinant adeno-associated vectors (rAAVs) designed to modulate the NPY system (Table 1).

**Table 1:** Comparison of different gene therapy strategies designed to modulate the NPY system, based on the use of recombinant adeno-associated vectors.

**First author (year)**	**Species**	**Model of epilepsy**	**Vector**	**Time of vector delivery**	**Transgene**
Richichi et al. (2004)	WT rats	Intrahippocampal and intracerebroventricular kainic acid; Kindling	rAAV2_NSE-NPY; rAAV1/2-NSE-NPY	Before seizure onset	Human pre-pro-NPY
Lin et al. (2006)	WT mice; Y1 -/- and Y2 -/- mice	Systemic kainic acid	rAAV1/2_NSE-NPY	Before seizure onset	Human NPY cDNA
Foti et al. (2007)	WT rats	Intraperitoneal kainic acid	rAAV2_CBA-NPY; rAAV2_CBA-NPY13-36	Before seizure onset	Full length and NPY13-36 (Species not specified)
Sørensen et al. (2008)	WT rats	None	rAAV1/2_NSE-NPY	N/A	Human pre-pro-NPY
Noè et al. (2008)	WT rats	Electrically induced status epilepticus	rAAV1/2_CBA-NPY	After seizure onset	Human pre-pro-NPY
Sørensen et al. (2009)	WT rats	Kindling	rAAV1/2_NSE-NPY	Before seizure onset	Human pre-pro-NPY
Noè et al. (2010)	WT rats	Intrahippocampal kainic acid	rAAV1_CBA-NPY; rAAV1/2_CBA-NPY	After seizure onset	Human pre-pro-NPY
Woldbye et al. (2010)	WT rats	Kindling; Subcutaneous kainic acid	rAAV1/2_NSE-NPY; rAAV1/2_NSE-Y2	After seizure onset	Human pre-pro-NPY Full length mouse Y2 receptor
Gøtzsche et al. (2012)	WT rats	Subcutanous kainic acid	rAAV1/2_NSE-Y5; rAAV1/2_NSE-NPY	Before seizure onset	Human pre-pro-NPY Full length mouse Y5 receptor
Olesen et al. (2012b)	WT mice	Subcutaneous kainic acid	rAAV1/2_NSE-Y1	After seizure onset	Full length mouse Y1 receptor
Olesen et al. (2012a)	WT mice	Subcutaneous kainic acid	rAAV1/2_NSE-Y5	After seizure onset	Full length mouse Y5 receptor
Dong et al. (2013)	WT rats	Intrahippocampal kainic acid	rAAV1/2_CMV-NPY	Before seizure onset	Full length NPY (species not specified)
Zhang et al. (2013)	WT rats	Intracerebroventricular kainic acid	rAAV1/2_NPY (unknown promoter)	Before seizure onset	Not specified
Nikitidou Ledri et al. (2016)	WT rats	Intrahippocampal kainic acid	rAAV1/2_NSE-NPY; rAAV1/2_NSE-Y2	Before seizure onset	Human pre-pro-NPY Full length mouse Y2 receptor
Powell et al. (2018)	GAERS (Genetic Absence Epilepsy Rats)	None	rAAV1/2_NSE-NPY	N/A	Human pre-pro-NPY
Melin et al. (2019)	WT rats	Intrahippocampal kainic acid	rAAV1_CAG-NPY/Y2	Before seizure onset	Human pre-pro-NPY Human Y2 receptor

Early attempts in this direction explored the anti-seizure potential of NPY overexpression mediated by rAAV serotype 2 (rAAV2) vector injection in the hippocampus (Richichi et al., 2004) or piriform cortex (Foti et al., 2007) in the rat kainate model of epilepsy. Importantly, Richichi et al. (2004) compared the effects of serotypes AAV2 and chimeric AAV1/2, both vectors with the human NPY gene driven by the neuron-specific enolase promoter (pNSE). A long-term transgene expression, confined in hilar interneurons, was observed with AAV2, while more widespread expression in diverse subtypes of neurons was observed with the AAV1/2 serotype, that also conferred a more robust protection from epileptogenesis and chronic seizures. Y1 or Y2 double knockout mice, contrary to the wild type, did not display any protection from seizure activity upon NPY gene therapy, indicating that activation of one (most likely Y2) or both of these receptor subtypes was essential for the NPY effect (Lin et al., 2006). More recently, the AAV1/2 expressing-NPY vector was infused into the thalamus or somatosensory cortex in a rat model of genetic generalized epilepsy (GAERS, Genetic Absence Epileptic Rats from Strasbourg), resulting in a reduced seizure activity, in particular when injected in the thalamus (Powell et al., 2018).

Some concerns on the potential for translatability to human application were raised by Sørensen et al. (2008). These authors claimed an impairment of synaptic plasticity and the attenuation of long-term potentiation of Schaffer collateral-CA1 synapses in naive rats upon unilateral vector injection in the hippocampus, with consequent deficits of hippocampal-based spatial discrimination learning (Sørensen et al., 2008). These unexpected findings were contrasted by the authors themselves in a following study that showed seizure protection with no impact on working memory performance tasks in kindled rats injected in both hippocampi with the AAV1/2-pNSE-NPY vector (Sørensen et al., 2009).

In any event, the initial attempts of NPY gene therapy had limited relevance for clinical translation: they were all carried out before epilepsy onset, in a scenario that is obviously non-reproducible in real patients and that did not take into account the aberrant changes occurring during epileptogenesis, which may significantly affect treatment effectiveness. In order to overcome this limitation, Noè et al. (2008) tested the effect of hippocampal injection of an AAV1/2 vector expressing NPY after the establishment of epilepsy in rats and found a decrease in seizure activity. Interestingly, this study also demonstrated preserved levels of Y2R into the AAV-injected hippocampus, with functional transport and high levels of release of the recombinant NPY to nerve terminals upon induction of neuronal depolarization. In a following report, the same authors delivered NPY using rAAV1, and observed a widespread transgene expression pattern throughout the injected hippocampi and a potent effect on seizure reduction, with no detectable evidence of immune response or cognitive impairment (Noè et al., 2010).

NPY is directly involved in the regulation of brain excitability by regulation of intracellular calcium and glutamate release, mainly through binding to and activation of Y1, Y2, and Y5 receptors (Berglund et al., 2003). As described above, whereas converging evidence supports an anti-epileptic role of Y2 (and to a lesser extent of Y5) receptors, the involvement of Y1Rs remains debated, with some evidence of pro-epileptic effects. Therefore, a simple increase in NPY levels may become a double-edged sword.

These considerations prompted alternative gene therapy strategies, oriented not only at increasing NPY secretion into the epileptic focus but, also, at re-shaping the NPY ligand-receptor system by the delivery of genes encoding for the different NPY receptors. To date, the only study performed to evaluate the effects of a brain overexpression of Y1 in an animal model of epilepsy indicates an increased susceptibility to kainate-induced seizures (Olesen et al., 2012b), consistent with the mentioned evidence of Y1R-mediated pro-epileptic effects (Gariboldi et al., 1998; Benmaamar et al., 2003). One study proved seizure reduction through the delivery in the rat hippocampus of an AAV pool of vectors for the concomitant expression of both Y5 and NPY (Gøtzsche et al., 2012), but no protective effect was observed with the AAV-Y5 vector alone (Gøtzsche et al., 2012; Olesen et al., 2012a). More robust and promising data have been obtained by overexpressing Y2 receptors, i.e., by seconding the adaptive up-regulation of these receptors observed in the epileptic tissue. Y2Rs proved to be sufficient to suppress acute seizures even when overexpressed alone, although the therapeutic outcome significantly increased in the case of concurrent treatment with an NPY expressing vector (Woldbye et al., 2010).

Attempts of combinatorial gene delivery have been accomplished by using two separate rAAV vectors (Nikitidou Ledri et al., 2016). This procedure, however, faces some limitations, such as an unknown transduction efficiency of the different vectors upon brain infusion or the potential obstacles that a heterogeneous viral pool could face in case of clinical application. In order to solve such issues, Melin et al. (2019) used an AAV1-based vector specially designed for the concurrent expression of both NPY and Y2 from a single viral construct, injected into both dorsal and ventral hippocampus to target the epileptogenic focus. This dual-gene vector delivery led to a detectable overexpression of both NPY and Y2R within the injected hippocampi, particularly pronounced into the dorsal CA1 and CA3 regions, and resulted in a remarkable decrease of EEG seizure frequency and duration in the kainic acid model of TLE (Melin et al., 2019).

## Problems and Opportunities

As described above, both NPY and its receptors display a high degree of complexity, from synthesis, processing and compartmentalized delivery or regulated secretion, to an intricated variety of biological effects, both at local and global circuit level. These elements have profound implications for gene therapy.

The majority of data reported in the literature derive from experiments performed with viral vectors constructed to express pre-pro-NPY (Table 1). In this context, the use of the full length NPY sequence may be advantageous, since it allows using the endogenous cellular machinery to process and pack the pro-peptide into vesicles, where the mature NPY is formed and then stored. In this way stimulus-dependent release of the peptide (e.g., at the onset of a seizure) can be preserved. Biosynthesis and stimulus-dependent release of mature NPY have been indeed shown *ex vivo* (Noè et al., 2008). While all this may occur in cells that physiologically express NPY, NPY gene delivery alone may not be sufficient for regulated release of the mature peptide in cells lacking/under-expressing one or more of the regulatory elements (e.g., processing enzymes, trafficking proteins) needed in such a complex multi-step system. One option to circumvent this problem could be linking the NPY gene sequence to the sequence of the laminar protein fibronectin (FIB), which induces a constitutive secretion as opposed to a regulated secretion (Foti et al., 2007). Finally, even after release, the effects of peptidases should be taken into account to understand and modulate NPY signaling.

The modulation of YRs expression requires an even more finely regulated sequence of events. Functional specificity of the NPY system depends largely on receptors. In this context, the processes of anterograde transport, internalization, recycling or degradation have been thoroughly characterized for only a few NPY receptors. These considerations lead to the suggestion that, if no specific cell targeting strategy is employed, gene therapy-induced overexpression of NPY or NPY receptors may be more efficient in (or even restricted to) cells that physiologically or pathophysiologically express them. The levels of released NPY and the coupling between ligand and receptor are also crucial for inducing the desired effect in the right cell target. It may be possible to obtain a certain degree of receptor selectivity by using, for example, N-terminally truncated forms of NPY (like NPY_3−36_ or NPY_13−36_ (Beck-Sickinger and Jung, 1995; Sajdyk et al., 2002; Foti et al., 2007; Pedragosa-Badia et al., 2013) that could favor Y2Rs dependent signaling.

## Outlook for Human Studies Using Viral Vector-Based Strategies

Despite this complexity, several anti-epileptic gene therapy strategies proved successful in modulating the inhibitory/excitatory balance within animal brain regions involved in seizure onset by focal overexpression of NPY alone or in combination with Y2 or Y5 receptors. Even if extended long-time studies to exclude side effects or neuropathological changes due to application of viral vectors still need to be performed (optimally in non-human primates), these compelling preclinical data may concretely prompt the design of a first-in-human gene therapy trial in drug-resistant epileptic patients. As an example, patients deemed suitable for surgical resection of a clearly mapped epileptogenic region may be enrolled in a first putative human study. This would allow to design a confined (and presumably more effective) transgene expression within the epileptogenic lesion only, while preserving the unaffected brain tissue and thereby lowering the risk of unpredictable side effects. In addition, should the treatment not prove to be effective or well-tolerated, patients would undergo resective surgery as originally planned.

Several issues should be taken into account in the study design. For example, hippocampal sclerosis, if extensive, may reduce vector diffusion and transduction efficacy, imposing a personalization of the dose. The choice of vector would largely depend on the strategy employed to regulate the expression of the therapeutic gene. As described in this review, all studies on gene therapy-mediated overexpression of NPY and/or its receptors in epilepsy models have been performed by using AAV vectors. However, the limited cargo capacity of AAVs may hinder their adaptability for clinical translation, in particular when complex regulatory mechanisms must be set in place. In fact, it would be desirable to regulate the levels of transgene expression in a patient-tailored manner, in response to endogenous and/or exogenous clues. While an endogenous control of the transgene expression system that responds to physiological stimuli (for example, glutamate accumulation) would be preferable, the time needed for the biosynthesis and delivery of the therapeutic protein(s) would be too long to arrest an ongoing seizure. Therefore, a more concrete alternative, although not applicable to the response to individual seizures, but rather on a general control of seizure threshold, may rely on the administration of external factors (i.e., specific molecules). These elements could selectively activate or inhibit transgene expression, by acting on specific regulatory sequences delivered along with the therapeutic gene cassette, in the same viral vector. This option, however, would require the exploitation of neurotropic vectors capable to host much larger exogenous DNA cargos, for example HSV derived vectors (Ingusci et al., 2019a,b).

Once the remaining gaps in knowledge and hurdles for gene therapy will be overcome, we may finally be able to treat epilepsy by acting on endogenous systems of neuromodulation. In a way, this is something that we may have already done, unconsciously and much less finely, with certain anti-epileptic drugs (Brill et al., 2006).

## Author Contributions

All authors listed have made a substantial, direct and intellectual contribution to the work, and approved it for publication.

## Conflict of Interest

The authors declare that the research was conducted in the absence of any commercial or financial relationships that could be construed as a potential conflict of interest.
